# Giant Cell Tumor of the Central Skull Base

**DOI:** 10.7759/cureus.69602

**Published:** 2024-09-17

**Authors:** Anuj Shah, Jacob Mesenger, Benjamin Schwartz, Gaurav Saigal, Sameer Samtani

**Affiliations:** 1 Department of Radiology, University of Miami Miller School of Medicine, Miami, USA; 2 Department of Radiology, University of Miami Health System/Jackson Memorial Hospital, Miami, USA

**Keywords:** clivus, giant cell tumor, magnetic resonance imaging, neurosurgery, pediatric oncology, skull base tumor

## Abstract

Giant cell tumor (GCT) of bone is an uncommon indolent tumor, typically occurring in the meta-epiphysis of long bones in young adults. GCT arising in the clivus is exceedingly rare, and even more uncommon in the pediatric population. We present a case of a 13-year-old patient diagnosed with a large GCT in the clivus. Initial clinical and radiographic findings were suspicious for a GCT, although other more common skull base tumors were also considered in the differential diagnosis. Certain key radiographic features suggested the diagnosis of a GCT, including the low T2 signal within the mass on MRI and the T2 hypointense rim corresponding with a cortical shell present on CT. An endoscopic biopsy confirmed the diagnosis. This report highlights the unique diagnostic challenges and broad differential in this case while underscoring the role of imaging in detection and precise anatomic delineation that helps guide therapeutic decisions and improve patient prognosis.

## Introduction

Giant cell tumors (GCTs) of bone are indolent neoplasms that constitute 5% of primary bone tumors and 20% of benign bone lesions in adults [1,2]. They can be locally aggressive and occasionally metastasize to the lung [3]. The tumors most often occur in patients between the ages of 20 to 40, and have a slight female predilection [1]. These tumors classically occur in the metaphysis or epiphysis of long bones following closure of the growth plate and are most commonly found in the distal femur, proximal tibia, and distal radius, with fewer than 20% of tumors occurring outside of these locations [1]. They are considered less common in children, with an incidence of between 1.8% and 10.6% of all new GCTs [4,5].

GCTs in the clivus are exceedingly uncommon and can be diagnostically challenging on imaging especially when they occur in the pediatric population. We present an unusual case of a GCT in the clivus of a pediatric patient.

## Case presentation

A 13-year-old female with no previous past medical history presented with a two-month history of numbness in the left V2 and V3 cranial nerve distribution as well as blurry vision in her right eye. This was accompanied by minimal facial swelling and occasional headaches. A few weeks later, she experienced a sudden onset of vision loss in the lower visual field of her right eye.

An MRI was obtained at an outside hospital, showing a large heterogeneously enhancing skull base lesion centered in the clivus, causing compression of the bilateral prechiasmatic optic nerves as well as invading the nasal sinuses. Imaging of the spine was normal.

On presentation to our center one month after her initial presentation, she was found to have markedly decreased vision in the right eye with only finger counting maintained. Vision was 20/25 in the left eye. The patient also had mild bilateral 6th nerve palsy and some nystagmus, particularly on upward gaze.

**Figure 1:**
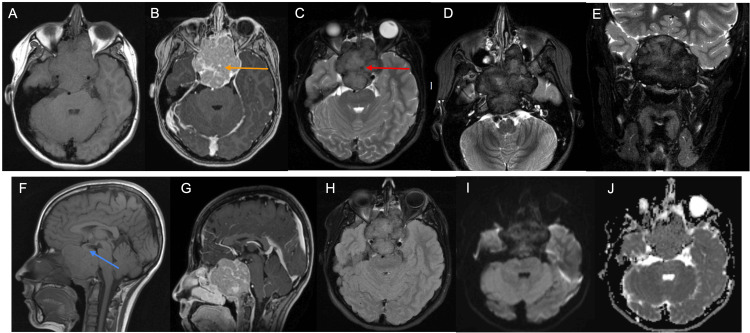
T1-weighted images in axial plane (A) before and (B) after gadolinium demonstrate a T1 isointense mass centered at the clivus with heterogeneous enhancement. (C) T2-weighted images in the axial plane at the same level demonstrate a T2 hypointense focus (red arrow) likely representing a flow void corresponding with the focus of enhancement on the postcontrast images (orange arrow). The T2-weighted images in the (D) axial plane at a more inferior slice and in the (E) coronal plane demonstrate diffuse hypointense signal within the mass and a marked T2 hypointense rim. T1-weighted images in the sagittal plane (F) before and (G) after gadolinium demonstrate superior displacement of the pituitary (blue arrow) without invasion. (H) FLAIR images in the axial plane demonstrate isointense to hypointense signal. (I) Diffusion-weighted and corresponding (J) ADC images demonstrate no diffusion restriction. FLAIR: Fluid-attenuated inversion recovery, ADC: Apparent diffusion coefficient.

MRI of the brain performed at our institution one month after the initial presentation demonstrated a large expansile T1 isointense, T2 hypointense, heterogeneously enhancing mass without diffusion restriction centered at the clivus with extension inferiorly into the nasopharynx (Figure 1). Superiorly, the mass displaced the sella and pituitary without invasion. Anteriorly, the mass extended into bilateral sphenoid sinuses, the posterior ethmoid air cells, and bilateral orbital apices with mass effect and partial encasement of bilateral prechiasmatic optic nerves. Anteriorly and superiorly, the mass expanded the planum sphenoidale, bulging into the anterior cranial fossa and causing a mass effect on the gyrus rectus. Laterally, the mass extended into the cavernous sinus with encasement of bilateral distal petrous and cavernous internal carotid arteries (ICAs) leading to mild narrowing of the right ICA flow void and no significant narrowing of the left ICA flow void. Dorsally, the mass extended into the prepontine cistern, compressing the ventral pons and partially encasing the basilar artery without significant narrowing. The mass demonstrated solid enhancement without cystic components. No fluid-fluid levels were noted. Foci of low T2 signal was noted within the mass, suggestive of flow voids corresponding with curvilinear enhancement on post-contrast sequences, likely representing prominent vessels. Additional foci of T2 hypointense signal were noted within the mass, corresponding with punctate calcifications on CT. Surrounding the mass was a marked T2 hypointense rim corresponding with a cortical rim seen on CT.

Based on these imaging findings, the initial differential diagnosis included aggressive sinonasal malignancies including rhabdomyosarcoma, primary and metastatic clival lesions, and less likely lymphoproliferative malignancy.

**Figure 2:**
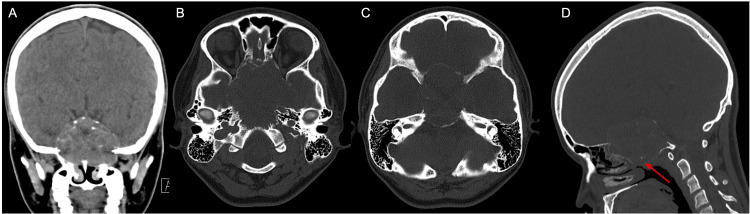
(A) CT images in coronal plane with soft tissue window show a slightly hyperdense soft tissue mass. (B) In the axial plane with bone window more inferiorly shows a lytic expansile mass with scalloping of the clivus and (C) more superiorly shows thinning of the bone without frank destruction. (D) The sagittal plane with bone window demonstrates the expansile lytic mass centered at the clivus containing hyperdense punctate foci likely representing punctate calcifications or residual bone (red arrow).

**Figure 3:**
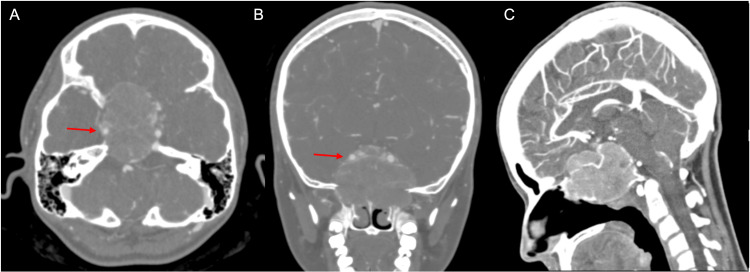
CT angiography images in (A) axial, (B) coronal, and (C) sagittal planes show mild narrowing of the right cavernous ICA (red arrows), arterial enhancement of the mass and multiple prominent vessels traversing the mass. ICA: Internal carotid artery.

Further characterization of the mass with a CT (Figure 2) and CT angiography (Figure 3) of the head was obtained, demonstrating an expansile slightly hyperdense lytic mass centered at the clivus with scalloping and thinning of the surrounding cortical bone. The mass appeared solid without any ground glass appearance or fluid-fluid levels. Notably, there was no frank bony destruction, aggressive periosteal reaction, or osseous matrix formation.

CT angiography of the head confirmed encasement of bilateral distal petrous and cavernous ICAs with mild narrowing of the right distal petrous ICA and cavernous ICA, and no significant narrowing of the left ICA (Figures 2, 3). There was moderate enhancement of the mass and multiple prominent vessels were seen traversing the mass.

Pathology 

**Figure 4:**
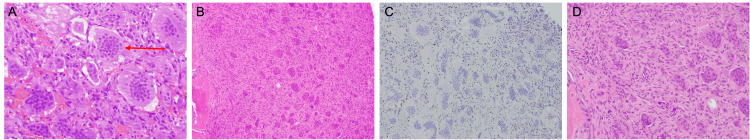
Hematoxylin- and eosin-stained pathology images of the tumor with (A) 200X and (B) 100X magnification, which reveals giant cell tumor of bone as evidenced by mixed multinucleated, osteoclast-like giant cells (red arrow) interspersed with mononuclear neoplastic cells. (C) 100X magnification image of immunohistochemistry showing mononuclear neoplastic cells positive for H3F3A G34W. (D) 200X image showing reactive bone formation and hemosiderin deposition.

An endoscopic biopsy of the mass was performed, which showed numerous non-neoplastic multinucleated, osteoclast-like giant cells scattered throughout the tumor with interspersed mononuclear neoplastic cells, as well as reactive bone formation and hemosiderin deposition (Figure 4). Immunohistochemistry revealed mononuclear neoplastic cells positive for H3F3A G34W, supporting the diagnosis of GCT of bone.

Management

**Figure 5:**
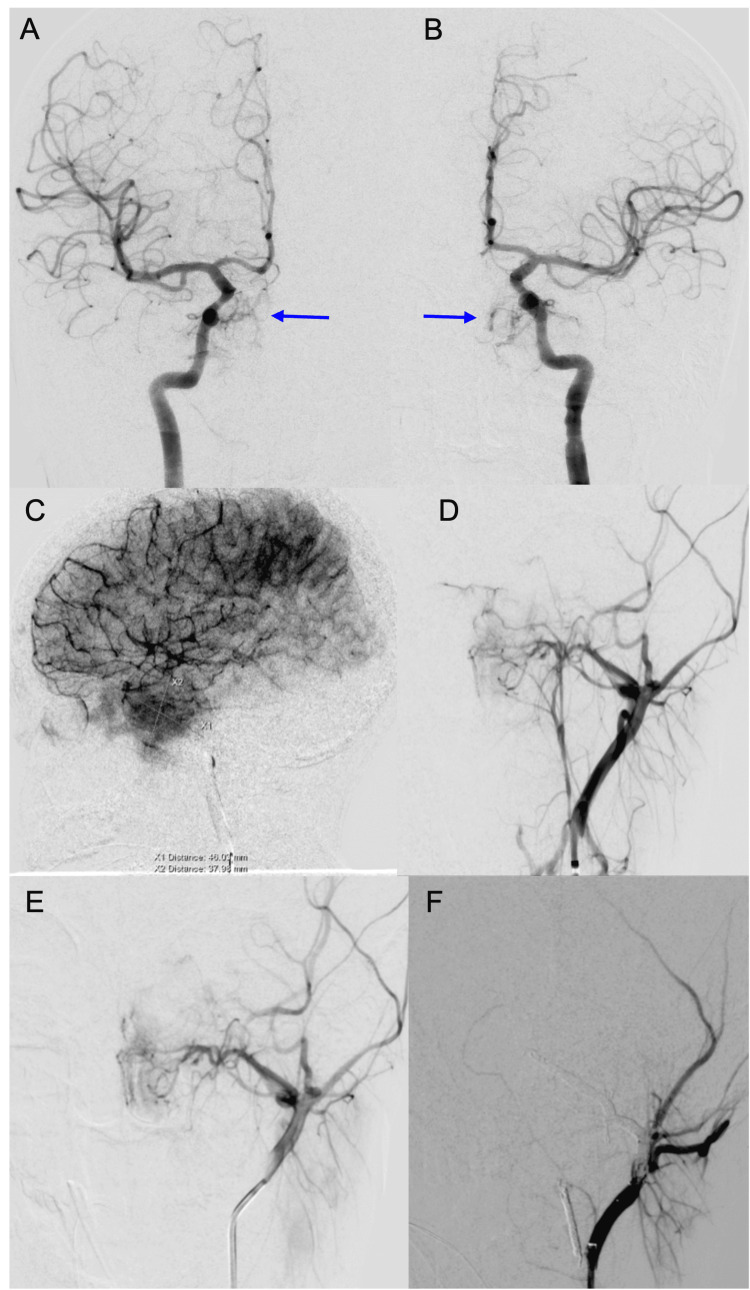
Angiogram of the (A) right ICA and (B) left ICA demonstrate branches of bilateral cavernous ICAs supplying the tumor (blue arrows). (C, D, E) Left ECA angiogram showing branches of the ascending pharyngeal and internal maxillary arteries supplying the tumor. Angiogram shows brisk filling of the left ECA branches without any evidence of fistula. There is hypervascularity within the tumor with capillary tumor blush. (F) Successful coil embolization of bilateral internal maxillary and left ascending pharyngeal arteries supplying the tumor in preparation for surgical resection. ICA: Internal carotid artery, ECA: External carotid artery.

Given the marked increased vascularity, the patient then underwent preoperative embolization of the left ascending pharyngeal artery and bilateral internal maxillary arteries (Figure 5) followed by partial transnasal transsphenoidal resection of the skull base tumor with bilateral maxillary antrostomies and partial ethmoidectomies. Resection was limited due to high tumor vascularity and the risk of extensive bleeding, and a large component of the tumor remained. However, following resection, the patient reported partial improvement in the vision in her right eye, but with residual limitations in the lateral and inferior visual field of her right eye. She also reported significant improvement in her facial numbness. She is expected to follow up with oncology for adjuvant treatment.

## Discussion

This case represents a unique occurrence of a GCT centered in the clivus of a pediatric patient. Previous literature on the occurrence of GCTs in the skull has shown that these tumors most often occur in temporal and sphenoid bones [3,6-8], and less commonly in the occipital bone, with only a handful of cases of GCTs centered at the clivus [9]. Even rarer is the involvement of the clivus in a pediatric patient, as seen in this case.

When a solitary bone tumor is observed to be involving the clivus in a pediatric patient, several common and uncommon entities must be considered. These include conditions such as fibrous dysplasia, Langerhans and non-Langerhans cell histiocytosis, lymphoma, chordoma, aneurysmal bone cyst, osteosarcoma, meningioma, brown tumor of hyperparathyroidism, plasmacytoma, giant cell reparative granuloma (GCRG), and metastases. Invasion of the clivus by way of extension from surrounding structures can also occur, as seen in tumors such as rhabdomyosarcoma, nasopharyngeal carcinoma, and invasive pituitary macroadenoma.

Of the aforementioned diagnoses, the primary ones to consider in a child of this age group would be fibrous dysplasia, aneurysmal bone cyst, invasive pituitary macroadenoma, lymphoma, Langerhans cell histiocytosis, osteosarcoma, rhabdomyosarcoma, giant cell reparative granuloma, and GCT. An aneurysmal bone cyst was excluded due to a lack of T2 hyperintense signal and the absence of fluid-fluid level [3,10]. A chordoma was considered less likely because of the low T2 signal within the mass signal and the lack of frank osseous destruction [3,10]. Osteosarcoma was considered unlikely because there was no osseous matrix formation or aggressive features such as bony destruction or periosteal reaction [3,10,11]. Rhabdomyosarcoma and nasopharyngeal carcinoma were also considered unlikely due to the lack of aggressive destruction of bone [3,12]. An invasive pituitary macroadenoma was excluded since the pituitary gland could be seen displaced superiorly by the mass. Langerhans cell histiocytosis was excluded because of the lack of bony destruction or other lytic lesions. Fibrous dysplasia of the skull base can be challenging to differentiate from a GCT and may require biopsy to do so. Fibrous dysplasia classically demonstrates low T1 and T2 signal on MRI and has a ground glass appearance on CT, although its appearance can vary greatly depending on the amount of mineralized matrix, fibrous tissue content, and cystic spaces [13,14]. Although primary skull base lymphoma is uncommon and may mimic multiple different skull base lesions, it typically involves more than one area of the skull base and demonstrates diffusion restriction, thus making it an unlikely diagnosis in this case [15]. GCRG appears identical to GCT, although they rarely involve the sphenoid bone [3,10,16]. Based on imaging appearance, which was suggestive of a primary bone tumor given a lack of fluid-fluid levels and lack of aggressive features, a diagnosis of GCT was considered as the primary diagnosis.

GCTs of bone often present on radiographs as lytic expansile lesions, eccentrically located within the metaphysis and epiphysis of long bones extending to the subchondral bone [11]. Classically, GCTs are well-defined lesions with cortical thinning, although they can sometimes be locally aggressive with cortical breakthrough [11]. Primary GCTs involving the clivus are extremely rare and infrequently reported in the literature [9]. MRI and CT are useful for evaluating the extension of the tumor into surrounding structures, as well as for evaluating underlying increased vascularity as in our case. The appearance of GCTs in the skull base can vary on MRI, although most prior cases have described low to intermediate signal intensity on T1- and T2-weighted images [11].

The tumor in this particular case was indeed isointense on T1 and hypointense on T2. The low T2 signal was likely due to hemosiderin deposition or fibrosis [3]. Additional foci of marked T2 hypointensity were seen within the tumor, some corresponding with curvilinear enhancement on post-contrast images suggestive of flow voids due to prominent vessels and others likely representing residual bone or calcifications, which are uncommon but can be seen in GCTs [17]. CT is the best modality for assessing osseous changes and the presence of calcifications. In this patient, a T2 hypointense rim was present at the margin of the tumor. It corresponded to the cortical shell present on CT with bony thinning and expansion of the surrounding cortical bone rather than osseous erosions and destruction, a key feature suggesting the diagnosis of GCT [11].

Surgery for intralesional curettage is the primary treatment for GCTs and generally controls up to 90% of the tumors [1]. Patients with larger, more aggressive, or recurrent disease may undergo wide excision, or in cases of unresectable tumors, moderate-dose radiation therapy [1]. In the case of this patient, only a small portion of the tumor was able to be surgically resected through the transsphenoidal approach, due to its spread into adjacent structures as well as its high vascularity. However, the amount of bleeding was able to be significantly reduced in this patient due to pre-operative embolization of the right and left internal maxillary arteries and left ascending pharyngeal artery.

## Conclusions

The development of a giant cell tumor (GCT) of bone in the region of the clivus in a pediatric patient represents a rare occurrence. One should keep this diagnosis in mind when suspecting a primary bone tumor with or without fluid levels centered at the clivus in a child. In this case, lack of fluid-fluid levels or aggressive features on imaging made a diagnosis of GCT of bone an initial possibility. This was further supported by the tumor’s T1 isointense and T2 hypointense signal on MRI.

Quick recognition of the suspected diagnosis provided the opportunity for early intervention. In particular, prompt recognition of rapid growth of the tumor, extension into surrounding vital structures, and increased vascularity on imaging preempted preoperative embolization and quick surgery, resulting in symptomatic relief to be followed by radiation and chemotherapy.
